# Pediatric New Daily Persistent Headache: Integrating Novel Research Methods to Support and Delineate Evolving Clinical Phenotypes

**DOI:** 10.3390/children13060743

**Published:** 2026-05-27

**Authors:** Hannah Rogan, Jenny John, Kevin Zhao, Scott Holmes, Alyssa A. Lebel

**Affiliations:** 1 Division of Pain Medicine, Department of Anesthesiology, Critical Care & Pain Medicine, Boston Children’s Hospital, Boston, MA 02115, USA; hannah.rogan@childrens.harvard.edu (H.R.); jenjohn@bu.edu (J.J.); kevin.zhao@childrens.harvard.edu (K.Z.); scott.holmes@childrens.harvard.edu (S.H.); 2 Department of Anesthesiology, Harvard Medical School, Boston, MA 02115, USA

**Keywords:** headache, new daily persistent headache, fMRI, MRI, machine learning

## Abstract

**Highlights:**

**What are the main findings?**

- There appear to be widespread cortical abnormalities in patients with new daily persistent headache (NDPH).
- Neuroimaging combined with artificial intelligence (AI) may sharpen definition of clinical headache disorders.

**What are the implications of the main findings?**

- AI and machine learning (ML) may enhance neuroimaging data in patients with NDPH.
- AI analysis of MRI data may offer a novel tool.

**Abstract:**

Pediatric new daily persistent headache (NDPH) is a clinically defined headache subtype that remains controversial due to a lack of unique and objective mechanistic features. For many headache subtypes, different, and sometimes unique, patterns of structural and functional changes can be observed in the brain, supporting a unique role for neuroimaging in identifying the presence and type of headache experienced. To date, there has been little research into pediatric NDPH and how it may have a unique mechanism relative to other headache subtypes. We review published research that addressed structural and functional neuroimaging in persons with NDPH. We found that research to date supports differences in both brain structure and function in persons with NDPH relative to healthy controls. Such differences reflect both cortical and sub-cortical regions of the brain. No studies to date have evaluated brain data between persons with NDPH and other headache subtypes. We discuss application of machine learning and artificial intelligence to validate NDPH as a unique headache diagnosis. We believe that future work pursuing both neuroimaging alongside machine learning can help inform the classification and differential diagnosis of pediatric patients with NDPH from other chronic headache conditions.

## 1. Introduction

New daily persistent headache (NDPH) is a debilitating disorder estimated to occur, as per recent studies, in 13–31% of pediatric patients with chronic daily headaches [1]. The IASP definition of NDPH includes a persistent (>3 months) headache that precipitously starts with a clearly remembered onset and continues in a non-remitting daily pattern. The precise etiology of this condition is poorly understood and may relate to the presentation of other conditions such as concussion and viral infection [2,3]. NDPH is an entity with an evolving definition, awaiting possible biomarker association.

Although NDPH has a unique identifier in terms of its rapid, unpredicted onset, it is currently defined solely by clinical factors and lacks objective support. Some authors have questioned the specific taxonomy of this chronic headache presentation and suggested including designated patients in the categories of chronic migraine and chronic pain disorders putatively due to central nervous system sensitization, such as complex regional pain syndrome and chronic pelvic pain. While some suggest NDPH is a subtype *of* conditions such as chronic migraine, others suggest NDPH is an entity *with* subtypes, given its features of migraine and tension-type headache. This has been reflected in the most recent iteration of the International Criteria for Headache Disorders (ICHD-III), which describes NDPH as having elements of either or both CM and TTH and also identifies two subgroups within NDPH: a self-limiting type and a refractory type. Regardless, there does appear to be a clear centralized component to NDPH that needs addressing. This is important, as no specific reported treatment modalities significantly affect headache pain in NDPH [3,4], and non-response of NDPH to medications effective in treating chronic migraine supports differential diagnosis [3]. There is a clinical need to both validate the unique nature of NDPH and identify the factors that make the condition a clinically unique entity, so patients can be accurately diagnosed and therefore effectively treated.

Clinical insight and patient reporting remain the most important source of data representing real-world experiences. However, the extent to which biological mechanisms can be outlined to support the presence of such clinical insight is not always appreciated. This may be due to a multitude of factors such as clinical or reporting bias, or limitations in technology, but nevertheless, it represents a problem faced by researchers attempting to delineate novel conditions to improve patient treatment services. This is an issue for headache research, as conditions such as post-traumatic headache and chronic migraine can be delineated, and yet, less frequently studied conditions such as new daily persistent headache struggle to obtain objective biomarkers.

The focus of this perspective is to discuss, using the limited published literature, the central impact on NDPH using both structural and functional neuroimaging approaches. We first provide a brief review of the general headache literature and then a broad summary of structural and functional neuroimaging research on NDPH. We then outline modern pathways through which we see this field progressing through the application of machine learning and AI initiatives and the success found from other headache cohorts.

## 2. General Headache

Chronic, primary headache is one of the most common disorders of the central nervous system [5] and contributes to significant disability across age groups [6]. Neuroimaging research in headache disorders has historically focused on chronic migraine, while fewer studies investigate other primary headache subtypes. Here, we briefly review the existing neuroimaging literature in various other primary headache disorders to contextualize findings reported in NDPH.

Across headache populations, neuroimaging findings are broadly distributed rather than localized to a single region, with alterations observed in multiple cortical and subcortical areas. Structurally, adult chronic headache patients demonstrate cortical thickness abnormalities in the left premotor, right primary somatosensory, and right prefrontal cortices, as well as default mode and executive control networks compared to controls [7]. Pediatric patients with migraine exhibit regional alterations as well, with gray matter (GM) volume reductions in the bilateral insula, motor/premotor, prefrontal, and cingulate cortices, right posterior parietal, and OFC [8]. Migraine—pediatric sample—with aura has been associated with increased GM volume in the left fusiform gyrus, while those without aura show reduced volume of this region [7]. Brain stem involvement has been found comparing adult persons with episodic migraine relative to controls, showing decreased hypothalamic volume in chronic migraine patients [9]. On a functional level, compared to both episodic migraine and controls, adult chronic migraine patients have increased functional connectivity (FC)—a measure of functional co-activation between two brain regions—between the bilateral habenula and salience network [10]. Increased FC has also been observed between the anterior cingulate cortex and right occipital gyri in chronic migraine compared to episodic migraine, suggesting a role for the occipital pole in chronification [11,12]. Notably, cohort differences are most frequently reported in brain regions involved in pain processing, emotional regulation, and large-scale functional networks [13,14]. As such, there is a strong indication that neuroimaging can assist in identification of biological mechanisms underlying headaches.

## 3. Structural Neuroimaging in NDPH

Multimodal studies have reported widespread cortical abnormalities in patients with NDPH. Due to the paucity of available studies, we present the findings as a whole group rather than differentiating adult versus pediatric cohorts which can be more clearly delineated from Table 1. Cortical thinning/reduced GM volume has been reported in left superior and middle frontal gyri, superior temporal gyrus, and fusiform gyrus. These findings may be associated with heightened pain sensitivity and, in some NDPH cases, more severe depressive symptoms [15,16]. In addition, increased GM volume in the left calcarine has been associated with increased symptoms of photophobia in patients with NDPH [15]. Compared to healthy controls, patients with NDPH showed reduced morphological similarity between the right superior frontal gyrus and the right hippocampus, as measured by the regional radiomics similarity networks, potentially suggesting disrupted structural integration between regions implicated in cognition and memory [17].

White matter (WM) disruptions have been often observed in individuals with NDPH, including reduced WM density and fractional anisotropy in the major frontal, temporal, and occipital tracts, including the bilateral inferior fronto-occipital fasciculus (IFOF), left superior longitudinal fasciculus (SLF), left inferior longitudinal fasciculus (ILF), right corticospinal tract (CST), and basal ganglia [17,18,19,20]. Increased radial and mean diffusivity in frontal, temporal, and occipital tracts may, in part, be due to potential demyelination or compromised WM integrity, possibly reflecting increased water molecule diffusion, which is often associated with tissue atrophy [17,20,21].

Regarding specific white matter tracts, reductions in neural density within the IFOF may suggest impaired information transfer from the occipital cortex, potentially reflecting axonal loss or damage. Research suggests this disruption may increase occipital cortex excitability, which could trigger cortical spreading depression and onset of headache pain [18]. The SLF is implicated in language processing and visuospatial attention [22]. A part of the SLF connects Broca’s area and Wernicke’s area, making the SLF integral for language function. Though patients with NDPH may not present with aphasia, declines in verbal memory and language skills have been reported compared to healthy controls [18]. Additionally, the SLF has been shown to contribute to the vestibular network function [23], and it is plausible that damage to this tract may lead to vertigo and dizziness often observed in patients with chronic headaches.

Characteristics of select NDPH structural imaging papers, including XYZ coordinates and contrasts (NDPH compared to healthy controls), can be found in Table 1. XYZ coordinates correspond to the brain regions listed in the table, and the contrast represents whether NDPH or HC exhibit increased or decreased structure in each region. When comparing adult versus pediatric populations (non-statistically), it would appear there are more broad changes to brain structure in the context of pediatric versus adult cohorts. However, there are two major limitations; first, pediatric cohorts experience concurrent brain changes relating to normal maturation, which makes it more likely to see group differences, and second, the very limited number of available studies to compare findings makes it likely that true group differences are not yet resolvable. Significant work remains to be able to comment as to differences in pediatric versus adult cohorts.

**Table 1:** Characteristics of select neuroimaging studies—structural imaging.

Author	Age Group	N	Description of Analysis	X	Y	Z	Contrast	Brain Regions
Naegel et al. 2022 [24]	Adults (18–69 years)	46	Voxel- and surface-based morphometry	41	−42	63	NDPH < HC	R somatosensory cortex
				−36	−8	−18	NDPH > HC	L hippocampus
Qiu et al. 2023 [16]	Pediatrics and adult (14–70 years)	65	Voxel- and source-based morphometry	−24.6	35.5	28.8	NDPH < HC	Rostral middle frontal gyrus
				−40.7	−72.1	−13.9	NDPH < HC	Fusiform gyrus
				−21	21	56	NDPH < HC	Cluster 1: superior frontal gyrus, middle frontal gyrus
				−24	−74	9	NDPH > HC	Cluster 1: calcarine
Li et al. 2024 [18]	Pediatrics and adult (14–60 years)	55	TBSS, surface-based analysis	38	24	−10	NDPH < HC (WM)	Cluster 1: R IFOF
				−41	−22	−18	NDPH < HC (WM)	Cluster 2: L ILF, L IFOF
				−52	−50	−9	NDPH < HC (WM)	Cluster 4: L SLF, L ILF
				22	−29	42	NDPH < HC (WM)	Cluster 1: R CST
				−35.6	1.8	51.4	NDPH < HC (GM)	L middle frontal cortex
				−54.5	−5.5	39.6	NDPH < HC (GM)	L precentral cortex
				−19.9	5.7	56.7	NDPH < HC (GM)	L superior frontal cortex
				29.8	23.5	3.1	NDPH < HC (GM)	R lateral orbitofrontal cortex and insula
Szabo et al. 2022 [15]	Pediatrics (12–18 years old)	26	Cortical analysis, subcortical analysis	48	−14	−4	NDPH < HC	R superior temporal gyrus
				−27	47	16	NDPH < HC	L rostral middle frontal gyrus
				−49	−6	−10	NDPH < HC	L superior temporal gyrus
				−19	−4	60	NDPH < HC	L superior frontal gyrus

Abbreviations: new daily persistent headache (NDPH); healthy control (HC); white matter (WM); gray matter (GM); left (L); right (R); inferior fronto-occipital fasciculus (IFOF); superior longitudinal fasciculus (SLF); inferior longitudinal fasciculus (ILF); corticospinal tract (CST); tract-based spatial statistics (TBSS).

## 4. Functional Neuroimaging in NDPH

Because of the low volume of available research studies, our summary presents findings collapsing across age cohorts, which can be more accurately delineated from Table 2. Diffuse functional connectivity (FC) alterations have been shown in patients with NDPH within bilateral, occipital, temporal, and frontal gyri, thalamus, cingulate, PAG (periaqueductal gray), VTA (ventral tegmental area), cerebellum, fusiform, lingual, and postcentral gyri, as well as other brainstem nuclei [25,26].

Decreased neuronal density has been identified in the left superior and middle frontal cortex, left precentral cortex, right lateral orbitofrontal cortex, and right insula. Such changes could reflect neuroadaptive changes within these regions, proposed to be possibly due to hyperexcitation leading to dendritic degradation [18].

Regarding brainwave frequencies, NDPH patients demonstrated higher global efficiency in the delta band, indicating more random, globally integrated network organization and possible hemispherical asymmetry associated with lateralized findings [26,27,28]. Reduced clustering in the left medial orbitofrontal cortex in the theta band has been seen in patients with depressive symptoms [25]. Additionally, elevated cortical activity in the ripple frequency band was observed across the whole brain, including both frontal and right temporal lobe [25]. In line with these findings, increased regional homogeneity and amplitude of low frequency fluctuations were reported in the left middle occipital gyrus in NDPH patients, further highlighting increased activity in visual processing regions [29].

Regarding lateralization, decreased cerebral blood flow and volume in various right hemisphere regions have been reported, despite the NDPH patient sample reporting bilateral headache pain [30]. Authors suggest this possibly indicates a compensatory vasoconstrictive response to ongoing headaches. Higher blood volume in the right orbital frontal cortex and high dentate nucleus (DN) GABA levels has been positively correlated with clinical anxiety scores. High cerebral blood flow and volume in the right thalamus have been positively correlated with length of headache episode, while higher DN and PAG levels [18] of glutamate/glutamine are negatively correlated with headache severity [30,31].

Characteristics of select NDPH functional imaging papers, including XYZ coordinates and contrasts (NDPH compared to healthy controls), can be found in Table 2. XYZ coordinates correspond to the brain regions listed in the table, and the contrast represents whether NDPH or HC exhibit increased or decreased functional connectivity in each region. From the data, it would appear there are more broad changes in functional connectivity in pediatric versus adult populations with NDPH. However, this must be interpreted with significant limitation, as there is insufficient data to date to provide meaningful conclusions relating to how adult vs pediatric populations differ with functional brain activity and connectivity in the context of NDPH. 

**Table 2:** Characteristics of select neuroimaging studies—functional imaging.

Author	Age Group	N	Description of Analysis	X	Y	Z	Contrast	Brain Regions
Szabo et al. 2022 [15]	Pediatrics (12–18 years old)	26	Functional connectivity analysis (seed-to-voxel whole-brain connectivity analysis)	56	−66	−4	NDPH < HC	R amygdala
				−18	−44	60	NDPH > HC	R hippocampus
				36	−10	−26	NDPH < HC	
				24	−38	−54	NDPH > HC	L hippocampus
				10	−60	46	NDPH < HC	L thalamus
				−26	12	−16	NDPH > HC	L cerebellum, crus 1
				28	20	0	NDPH > HC	
				−48	26	10	NDPH > HC	R cerebellum, crus 2
				−32	−48	4	NDPH < HC	
				44	−64	54	NDPH > HC	L cerebellum, crus 2
				40	−78	12	NDPH < HC	R cerebellum 3
				48	20	42	NDPH > HC	L cerebellum 7
				−16	−46	16	NDPH > HC	L cerebellum 9
				−50	−72	32	NDPH > HC	R cerebellum 10
				18	−64	−50	NDPH < HC	L cerebellum 10
Wang et al. 2023 [25]	Adults	66	Region of interest (ROI)-based analysis	12	−63	6	NDPH < HC	Seed: R lingual G; Cluster 1: R calcarine, R fusiform G, L calcarine
				18	−81	0	NDPH < HC	Seed: L sup OG; Cluster 1: R lingual G, R fusiform G
				21	−45	3	NDPH < HC	Seed: R middle OG; Cluster 1: R lingual G, L lingual G, R calcarine, R fusiform G
				3	45	51	NDPH > HC	Seed: L inferior OG; Cluster 1: R sup frontal G, medial
				45	−78	6	NDPH < HC	Seed: L inferior OG; Cluster 1: L lingual G, L fusiform G, L inferior OG
				39	−84	0	NDPH < HC	Seed: L inferior OG; Cluster 2: R middle OG, R lingual G, R inferior OG
				−12	−78	12	NDPH < HC	Seed: R inferior OG; Cluster 1: L lingual G, L fusiform G, L inferior OG
				21	−45	3	NDPH < HC	Seed: R inferior OG; Cluster 2: R lingual G, R fusiform G
				27	−66	−9	NDPH < HC	Seed: R fusiform G; Cluster 1: R/L lingual G; R inferior OG, R calcarine
				15	−81	39	NDPH < HC	Seed: R fusiform G; Cluster 2: R cuneus, R sup OG
				45	−18	33	NDPH < HC	Seed: L postcentral G; Cluster 1: R pre- and postcentral G
				−42	−12	48	NDPH < HC	Seed: R postcentral gyrus; Cluster 1: L post- and precentral gyrus
				30	−27	60	NDPH < HC	Seed: R postcentral gyrus; Cluster 2: R precentral gyrus
				−6	−90	18	NDPH > HC	Seed: R thalamus; Cluster 1: L/R cuneus, L calcarine, R superior OG
				42	0	18	NDPH < HC	Seed: R superior temporal G; Cluster 1: R postcentral G, R Rolandic operculum
Wang et al. 2024 [32]	Adults	66	ROI to whole-brain voxels	15	−48	−42	NDPH < HC	Seed: iMRt_R; Cluster 1: R Cerebellum_9
				48	−21	0	NDPH < HC	Seed: mRt_L; Cluster 1: R temporal sup
				−9	−66	−30	NDPH < HC	Seed: LC_L, Cluster 1: L Cerebellum_Crus2
				−3	−57	−39	NDPH < HC	Seed: LC_R; Cluster 1: L Cerebellum_Crus2, R Cerebellum_9
				−3	−54	−12	NDPH < HC	Seed: LDTg_CGPn_L; Cluster 1: L Cerebellum_4_5, R Vermis_4_5
				−3	−20	−27	NDPH < HC	Seed: LDTg_CGPn_R; Cluster 1: L Cerebellum_Crus1, L Cerebellum_6, L Cerebellum_Crus2, L Vermis_4_5, L Vermis_6, R Cerebellum_Crus2
				−12	−81	−48	NDPH < HC	Seed: MnR; Cluster 1: L Cerebellum_Crus2
				−15	−18	3	NDPH < HC	Seed: MnR; Cluster 2: L Thal_VPL, L Thal_VL
				−18	−66	30	NDPH < HC	Seed: MnR; Cluster 3: L precuneus, L cuneus
				−9	−15	27	NDPH < HC	Seed: MnR; Cluster 4: R/L Cingulate_Mid
				−30	−72	−33	NDPH < HC	Seed: MPB_L; Cluster 1: L Cerebellum_Crus1, L Cerebellum_Crus2
				0	−24	12	NDPH > HC	Seed: PAG; Cluster 1: L/R Thal_PuM
				−3	−27	−6	NDPH < HC	Seed: PAG; Cluster 1: R Temporal_Sup, R Insula
				0	0	24	NDPH < HC	Seed: PAG; Cluster 2: R/L Cingulate_Mid
				−36	9	−9	NDPH < HC	Seed: PAG; Cluster 3: L Temporal_Sup, L Insula
				6	−12	27	NDPH < HC	Seed: VTA_PBP_L; Cluster 1: L Cinulgate_Mid, R Thal_IL
				−30	−56	−36	NDPH < HC	Seed: VTA_PBP_L; Cluster 2: L Cerebellum_Crus1, L Cerebellum_Crus2, L Cerebellum_7b
				−6	−33	−12	NDPH < HC	Seed: VTA_PBP_L; Cluster 3: R Thal_VPL
				−27	−69	−36	NDPH < HC	Seed: VTA_PBP_R; Cluster 1: L Cerebellum_Crus1, L Cerebellum_Crus2
				9	−15	−15	NDPH < HC	Seed: VTA_PBP_R; Cluster 2: R SN
Zhang et al. 2023 [29]	Adults	60	ReHo, ALFF, and seed-based to whole-brain FC	−30	−99	3	NDPH > HC	ReHo, Cluster 1: MOG_L
				−27	−102	3	NDPH > HC	ALFF, Cluster 1: MOG_L
				−27	−102	3	NDPH > HC	ALFF, Cluster 1: MOG_L

Abbreviations: new daily persistent geadache (NDPH); healthy control (HC); left (L); right (R); gyrus (G); occipital gyrus (OG); inferior medullary reticular formation (iMRt); mesencephalic reticular formation (mRt); locus coeruleus (LC); laterodorsal tegmental nucleus–central gray of the rhombencephalon (LDTg_CGPn); median raphe (MnR); ventral posterolateral thalamus (Thal_VPL); ventral lateral thalamus (Thal_VL); pulvinar medial thalamus (Thal_PuM); intralaminar thalamus (Thal_IL); middle cingulate gyrus (Cingulate_Mid); superior temporal gyrus (Temporal_Sup).

## 5. Machine Learning as a Future Direction

The prospect of identifying NDPH as a unique clinical entity will likely be based on efforts in machine learning and artificial intelligence. Both ML and AI have been applied in prior work in the context of headache as it relates to diagnostics, predicting clinical trajectory, and forecasting upcoming headache attacks; in this regard, they are used to predict treatment effects, to uncover hidden information, and for remote monitoring and headache management [33]. Notably, to date, there have been no machine learning efforts inclusive of new daily persistent headache, as shown by a recent review focused on the use of ML/AI in headache [34].

To address the paucity of the available literature, emerging technologies validated in other cohorts offer new insight, particularly as it relates to leveraging existing data and models. That is, prior work has shown that data augmentation strategies can be applied when evaluating migraine and sub-categories of the condition [35]. This work did not use neuroimaging approaches but showed that by using data augmentation procedures in conjunction with models such as support vector machines, accuracies of sub-cohort classifications (e.g., migraine with and without aura) can be 95% and above. The application of data augmentation strategies in brain imaging has received little attention. In the context of brain tumor segmentation, methods such as random rotation, conditional GAN, TensorMixup, and others have shown potential in augmenting small data sets to reach capacity for larger ML-based algorithms to be applied, producing highly heterogeneous results [36] Notably, such techniques require initial sample inputs that often stress neuroimaging-based work.

An alternative is to use models developed on cohorts that have similar features to NDPH. That is, using forms of transfer learning, models can be used to predict outcomes and clinical features from novel data sets that are trained on independent data sets, similar to external validation. Intentionally building models on more abstract features permits more broad cohort application of models [37] but is intentionally limited to permit cross-application. To date, this has not been explored in the context of NDPH or headache but may offer preliminary insight into more abstract clinical features. Based on the complexities associated with brain imaging, it is likely this approach faces significant challenges; however, prior findings in persons with post-traumatic headache show that brain imaging and behavioral/clinical data contribute greatly towards cohort classification [38], supporting the pursuit of brain imaging as an adjunct to clinical diagnostics. There have been a number of examples where fMRI (resting state functional connectivity, REHO, ALFF) has been used in conjunction with different ML-based algorithms, such as long short-term memory models and convolutional neural networks, to support diagnostic accuracies of 90% and above (see [39] for review). The application of machine learning, both through data augmentation and transfer learning approaches, may offer insight into unique clinical cohorts such as NDPH.

## 6. Limitations

There are several limitations that were encountered throughout this manuscript. In the NDPH published literature, the authors only provided a summary; we did not dive into the risk of bias and quality of each study, so caution should be taken in the interpretation of findings. Though this manuscript is written from the pediatric perspective, the current literature has minimal pediatric representation. Adult findings can be considered when comparing to pediatrics, but findings cannot be generalized across age groups due to age-related neurobiological differences. Other limitations of the current literature include small sample sizes, cross-sectional designs, heterogenous age ranges, lack of replication, absence of direct comparisons with other headache subtypes, differing analytic pipelines with variable combinations of AI models and algorithms, limited acceptance of AI applications by the medical community, and uncertain clinical generalizability. These limitations directly constrain the conclusions that can be reached and inhibit the extent of AI/ML applications to the data.

**Figure 1:**
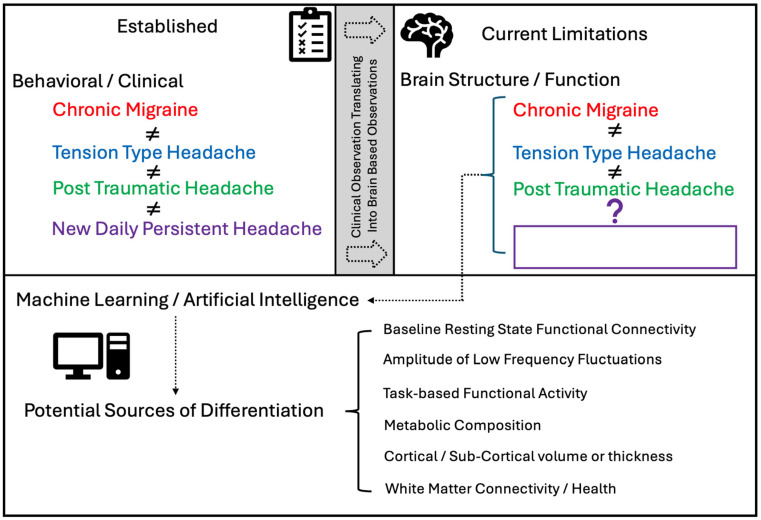
Proposed schematic pipeline for NDPH characterization with AI/machine learning. This is a schematic demonstrating how current observations relating to how NDPH can be resolved relative to other forms of headache using behavioral and clinical data has not been accomplished using brain imaging data. These efforts will likely be supported by machine learning/AI initiatives and be realized using whole-brain approaches that integrate one or multiple brain imaging modalities. To date, not enough data has been acquired to understand which features will likely produce the most important factors that will differentiate NDPH from other headache conditions.

Due to the paucity of the literature, the authors also could not propose a mechanistic model for the development of NDPH in the brain that was based on such studies. While such a mechanism is well established in conditions such as chronic migraine in pediatrics, the available data is such that a framework in NDPH would not be evidence-based. The evidence is not yet robust enough to draw conclusions regarding known headache neuroimaging patterns in other conditions, such as involvement of the default mode network. While the literature to date can certainly be considered when comparing NDPH to other chronic headache subtypes, direct conclusions cannot yet be drawn. However, to address this gap, Figure 1 introduces a dynamic framework the authors created to propose the categorical similarities and differences between headache subtypes that can eventually be used to identify NDPH as a unique entity comparatively. As we continue to grow the evidence base of imaging in NDPH, AI/ML may be utilized to push efforts forward and analyze the literature in broader and more innovative ways.

## 7. Conclusions

Findings from this review address a modern challenge in clinical neuroscience relating to patient classification. Limited neuroimaging research in NDPH, particularly the pediatric population, increases the difficulty of finding objective identifiers to define it as a distinct diagnosis. Without a robust array of the literature, conclusions cannot yet be drawn using imaging in NDPH compared to other headache subtypes. As we evolve our understanding of neuroscience and seek more precise delineation of clinical conditions such as NDPH, we must also evolve our methods to support more efficient pathways for disease detection and clinical implementation. The future of such methods may involve the integration of AI and ML with imaging, biochemical, and clinical data. Significant research remains to be performed in this area prior to clinical implementation. Although AI and ML are likely to play increasingly prominent roles in this process, it is critical that clinical insight and validation remain fundamental in this evolving landscape.

## Data Availability

No new data were created or analyzed in this study.

## Abbreviations

The following abbreviations are used in this manuscript:

NDPH	New daily persistent headache
AI	Artificial intelligence
ML	Machine learning
MRI	Magnetic resonance imaging
IASP	International Association for the Study of Pain
GM	Gray matter
OFC	Orbitofrontal cortex
FC	Functional connectivity
WM	White matter
IFOF	Inferior fronto-occipital fasciculus
SLF	Superior longitudinal fasciculus
ILF	Inferior longitudinal fasciculus
CST	Corticospinal tract
PAG	Periaqueductal gray
VTA	Ventral tegmental area
DN	Dentate nucleus
fMRI	Functional magnetic resonance imaging
REHO	Regional homogeneity
ALFF	Amplitude of low-frequency fluctuation
HC	Healthy control
